# Supplementary material for: Vagus nerve stimulation boosts the drive to work for rewards
Source: Nat Commun. 2020 Jul 16;11:3555. doi: 10.1038/s41467-020-17344-9 (PMC7366927; doi:10.1038/s41467-020-17344-9)
Supplement: Supplementary file 1 — Supplementary Information [file 41467_2020_17344_MOESM1_ESM.pdf]

Supplementary Information:

Vagus nerve stimulation boosts the drive to work for rewards

Monja P. Neuser[1], Vanessa Teckentrup[1], Anne Kühnel[1,2],

Manfred Hallschmid[3-5], Martin Walter[1,6,7], & Nils B. Kroemer[1*]

1 Department of Psychiatry and Psychotherapy, University of Tübingen, Calwerstraße 14, 72076 Tübingen, Germany
2 Department of Translational Research in Psychiatry, Max Planck Institute of Psychiatry and International Max Planck Research School for Translational Psychiatry (IMPRS-TP), Kraeplinstraße 2-10, 80804 Munich, Germany
3 Department of Medical Psychology and Behavioral Neurobiology, University of Tübingen, Otfried-Müller-Straße 25, 72076 Tübingen, Germany
4 German Center for Diabetes Research (DZD), Otfried-Müller-Straße 10, 72076 Tübingen, Germany
5 Institute for Diabetes Research and Metabolic Diseases of the Helmholtz Center Munich at the Eberhard Karls University Tübingen, Otfried-Müller-Str. 10, 72076 Tübingen, Germany
6 Otto von Guericke University Magdeburg, Department of Psychiatry and Psychotherapy, Leipziger Straße 44, 39120 Magdeburg Germany
7 Department of Psychiatry and Psychotherapy, University Hospital Jena, Philosophenweg 3, 07743 Jena, Germany

Corresponding author*

Dr. Nils B. Kroemer, nils.kroemer@uni-tuebingen.de

Calwerstr. 14, 72076 Tübingen, Germany

Supplementary Methods

During the sessions, we measured pulse, weight, and height as well as waist and hip circumference according to the recommendations of the World Health Organization[1]. Moreover, participants reported their last meal and drink and female participants further reported oral contraceptive use as well as the beginning of their last menstrual cycle. Pulse was also measured after the effort task.

At three time points, participants also responded to state questions presented on a computer screen as VAS using the joystick on an Xbox-360 controller (Microsoft Corporation, Redmond, WA). Items included questions on metabolic state (hunger, fullness, thirst) and mood, which were derived from the Positive and Negative Affect Schedule (PANAS[2]).

After completing the task block and a repetition of the state VAS, participants received their breakfast and a snack according to the food reward (energy) points earned. We provided a bowl of cereals for breakfast and a snack, both resuming the food reward (energy) points earned during the effort task: First, 100 ml of milk (or almond milk as vegan/lactose free alternative; ~68 kcal) were deducted from the total earned energy points. Second, participants could choose between three different chocolate bars (Twix, Mars or Snickers sticks; ~100 kcal each, Mars Inc., McLean, VA). The remaining points were converted into a serving of cereal. Since several participants had only earned few energy points, they received no additional snack and the volume of the milk was reduced to match the volume of the earned cereal. Participants received the bowl for breakfast and were instructed that this was their food reward and that they could eat as much as they liked. A 10-min break for breakfast was scheduled, but most of the participants finished eating before the end of the break.

For block-wise comparison across trials, absolute frequencies of button pressing, F_{BP}, were linearly transformed into relative frequency values at each time point *t*, F_{r_t} (Equation 1), by dividing absolute frequencies by the individual maximum frequency, $F_{\max}$, estimated during the training and practice in Session 1 (see Methods):

$$F_{\mathrm{r}_t} = \frac{F_{\mathrm{BP}_t} * 100}{F_{\max(\mathrm{S1})_i}} \tag{1}$$

To dissociate effort and rest phases, we segmented the effort trajectories (time-level data) into work and rest segments. The criteria for segment boundaries were based on the first temporal derivative of F_{r_t}. Work segment onsets were defined as a positive slope at *t*, combined with a cumulative increase of at least 10 units and no element smaller than -20 during the next second (Equation 2). Rest segment onsets were defined by inspection of the density distribution of the first temporal derivative of F_{r_t}. We determined the local minimum of the distribution on the group level, which indicated a visible distinction between effort and rest phases at a value of ~-20 (2.8 *SD)* for both levels of task difficulty (Equation 3).

$$\mathrm{ONSET_e} = \left(\frac{d\,F_{\mathrm{r}_t}}{d\,t} > 0\right) \wedge \left(\sum_{t+9}^{t} \frac{d\,F_{\mathrm{r}_t}}{d\,t} \geq 10\right) \wedge \left(\forall x \in \left[\frac{d\,F_{\mathrm{r}_t}}{d\,t}; \frac{d\,F_{\mathrm{r}_{t+9}}}{d\,t{+}9}\right] : \neg(x < -20)\right) \tag{2}$$

$$\mathrm{ONSET_r} = \frac{d\,F_{\mathrm{r}_t}}{d\,t} < -20 \tag{3}$$

For each work segment, we computed an invigoration slope (S_{I}) from segment onset to its first local peak. Peaks were determined using the MATLAB function findpeaks with default settings (cf. Fig. 1 for illustration). If no peak was found by the algorithm, the endpoint of the slope was set to the first data point of a plateau (second derivative = 0) or, alternatively, to the maximum F_{r_t} value in the segment.

To predict invigoration (S_INVSLO), we used a two-level hierarchical mixed-effects model as defined in HLM (for detailed results, see Supplementary Table 1). To predict effort maintenance, we predicted average relative frequency instead (for detailed results, see Supplementary Table 2). I_ indicates interaction terms (DRM = Difficulty × Reward Magnitude, DIFF = difficulty, REWM = reward magnitude, S = STIMCOND),

Level-1 Model:

$S\_INVSLO_{ti} = \pi_{0i} + \pi_{1i}*(STIMCOND_{ti}) + \pi_{2i}*(FOOD_{ti}) + \pi_{3i}*(REWM_{ti}) + \pi_{4i}*(CDIFF_{ti}) + \pi_{5i}*(I\_DRM_{ti}) + \pi_{6i}*(I\_SDIFF_{ti}) + \pi_{7i}*(I\_SREWM_{ti}) + \pi_{8i}*(I\_SFOOD_{ti}) + \pi_{9i}*(I\_SDRM_{ti}) + e_{ti}$

Level-2 Model:

$\pi_{0i} = \beta_{00} + \beta_{01}*(ORDER_i) + \beta_{02}*(STIMSIDE_i) + r_{0i}$

$\pi_{1i} = \beta_{10} + \beta_{11}*(ORDER_i) + \beta_{12}*(STIMSIDE_i) + r_{1i}$

$\pi_{2i} = \beta_{20} + \beta_{21}*(ORDER_i) + \beta_{22}*(STIMSIDE_i) + r_{2i}$

$\pi_{3i} = \beta_{30} + \beta_{31}*(ORDER_i) + \beta_{32}*(STIMSIDE_i) + r_{3i}$

$\pi_{4i} = \beta_{40} + \beta_{41}*(ORDER_i) + \beta_{42}*(STIMSIDE_i) + r_{4i}$

$\pi_{5i} = \beta_{50} + \beta_{51}*(ORDER_i) + \beta_{52}*(STIMSIDE_i) + r_{5i}$

$\pi_{6i} = \beta_{60} + \beta_{61}*(ORDER_i) + \beta_{62}*(STIMSIDE_i) + r_{6i}$

$\pi_{7i} = \beta_{70} + \beta_{71}*(ORDER_i) + \beta_{72}*(STIMSIDE_i) + r_{7i}$

$\pi_{8i} = \beta_{80} + \beta_{81}*(ORDER_i) + \beta_{82}*(STIMSIDE_i) + r_{8i}$

$\pi_{9i} = \beta_{90} + \beta_{91}*(ORDER_i) + \beta_{92}*(STIMSIDE_i) + r_{9i}$

ORDER and STIMSIDE have been centered around the grand mean.

To further characterize the decision to maintain effort or take a break, we applied a previously described cost-evidence accumulation model[3, 4, 5]. In this model, the duration of work and rest segments is used to fit an average amplitude of cost evidence as well as cost-evidence accumulation and dissipation slopes across all work segments. More specifically, the duration of a work segment (TE) is defined as follows:

$$\mathrm{TE} = \frac{A}{\mathrm{SE}} \tag{4}$$

and the duration of rest segment (TR):

$$\mathrm{TR} = \frac{A}{\mathrm{SR}} \tag{5}$$

where *A* is the shared amplitude of cost-evidence variations and SE and SR are cost-accumulation and cost-dissipation slopes, respectively. Differences in difficulty or reward magnitude could in principle affect all three parameters of the model and are incorporated as linear combinations for each parameter. However, for the current analysis, we used a reduced set of free parameters that were previously reported after extensive model comparisons [3, 4, 5]. Moreover, we set the mean amplitude, *A*, to 1 and introduced an additive taVNS effect for each parameter to estimate taVNS-induced changes leading to the following equations:

$$A = 1 + (A_{\mathrm{reward}} + A_{\mathrm{reward}_{\mathrm{taVNS}}} * \mathbf{Stim}) * \mathbf{R} \tag{6}$$

$$\mathrm{SE} = (\mathrm{SE}_{\mathrm{mean}} + \mathrm{SE}_{\mathrm{mean}_{\mathrm{taVNS}}} * \mathbf{Stim}) + (\mathrm{SE}_{\mathrm{diff}} + \mathrm{SE}_{\mathrm{diff}_{\mathrm{taVNS}}} * \mathbf{Stim}) * \mathbf{R} \tag{7}$$

$$\mathrm{SR} = (\mathrm{SR}_{\mathrm{mean}} + \mathrm{SR}_{\mathrm{mean}_{\mathrm{taVNS}}} * \mathbf{Stim}) + (\mathrm{SR}_{\mathrm{reward}} + \mathrm{SR}_{\mathrm{reward}_{\mathrm{taVNS}}} * \mathbf{Stim}) * \mathbf{R} \tag{8}$$

Here, the mean parameters are the average across all segments and the reward or difficulty parameters modulate the slopes or intercept in the corresponding trials. **R** and **D** are vectors containing the effect-centered reward and difficulty levels of each segment. **Stim** is a vector indicating for each segment if taVNS was administered (1) or sham (0). Hierarchical Bayesian models were fit using Markov chain Monte Carlo sampling (MCMC) implemented in JAGS. Here, participant level parameters are drawn from group-level distributions for each parameter. Group-level priors were flat normal distributions for all parameter means (*M* = 0, *SD* = 32). We sampled from 3 chains with 5.000 burn-in samples and 12.500 samples for each chain. In line with previous

studies, reward magnitude increased the dissipation slope, indicating reduced resting duration in the high reward condition (Supplementary Table 3). As we did not find conclusive evidence for the previously described modulating effect of reward magnitude on the amplitude or of difficulty on the effort accumulation slope, we additionally fit the full model including all possible moderators to the data. However, the model did not converge, indicating overspecification of the model. Nonetheless, credible intervals of all modeled taVNS effects included 0 even in the case of none convergence, indicating that we did not miss any potential taVNS effects in the reduced model.

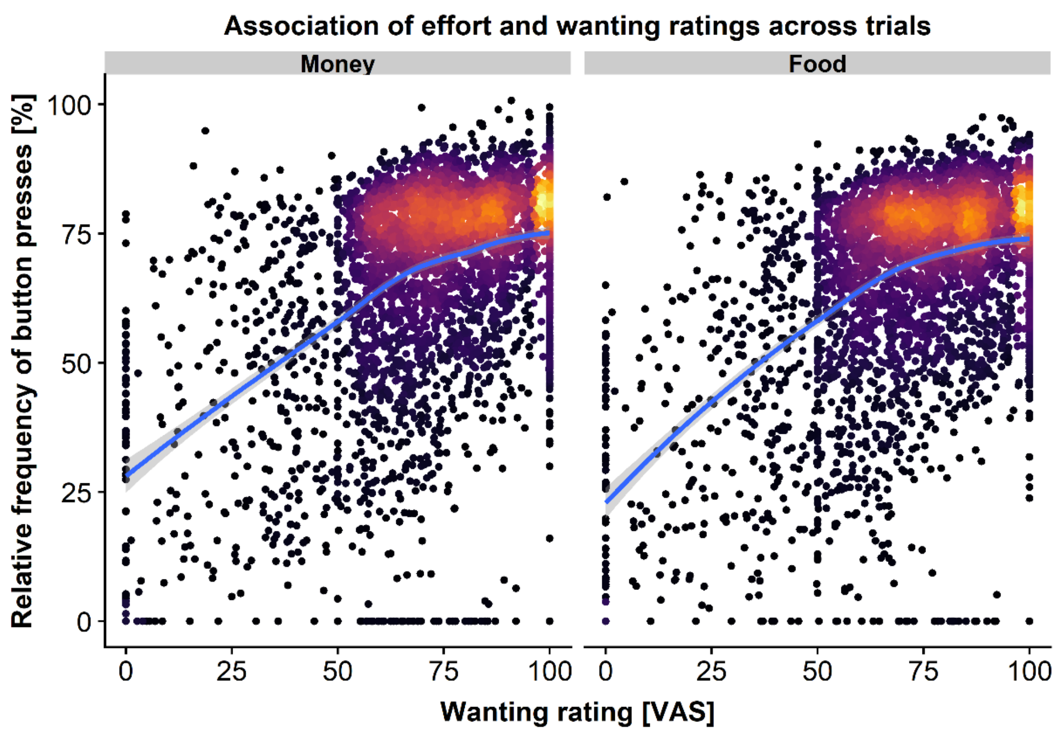

Supplementary Figure 1. Comparable association of subjective wanting ratings and effort maintenance for money and food rewards. Each dot corresponds to one trial (n = 7776 trials of 81 participants) and lighter colors show greater density of observations. The blue line reflects a generalized additive model smoothing fit line. Error bands (gray shading) depict the 95% confidence interval of the mean as estimated at the trial level; VAS = visual analog scale. Source data are provided as a Supplementary Source Data file.

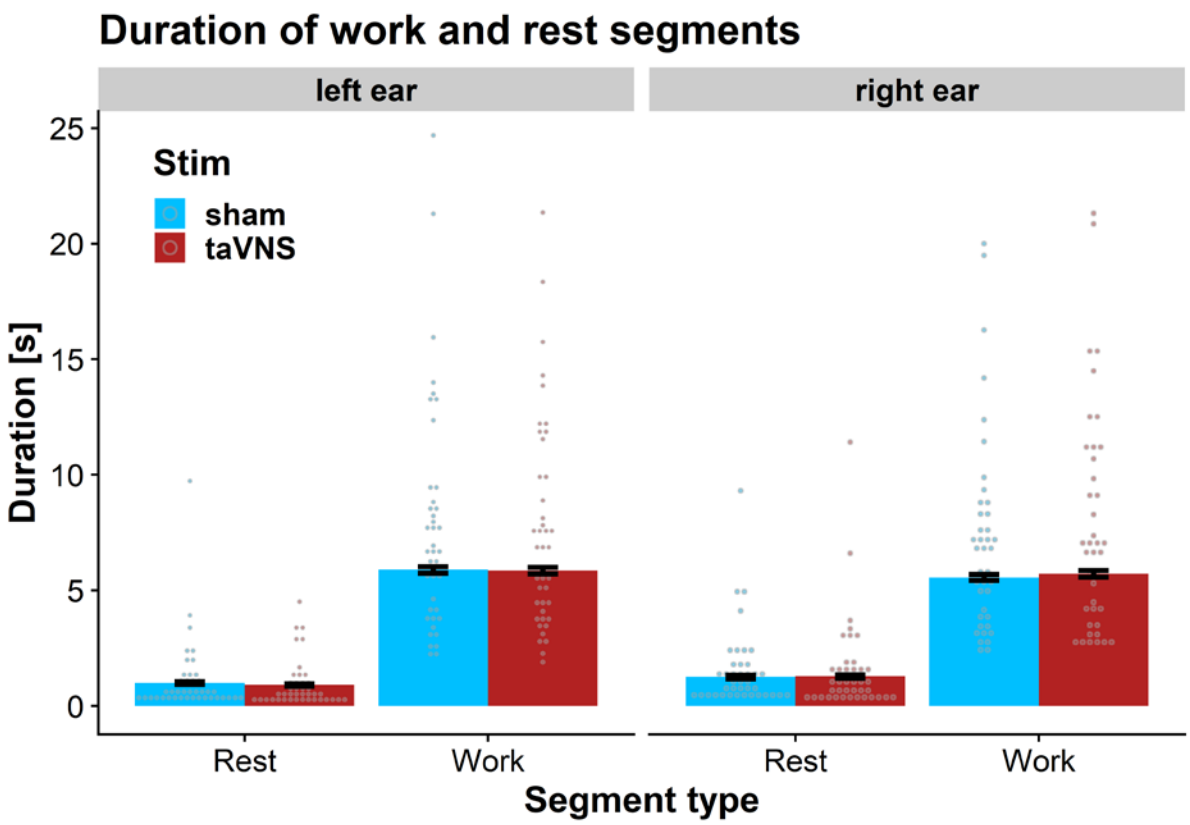

Supplementary Figure 2. No differences in work and rest durations between transcutaneous auricular vagus nerve stimulation (taVNS) and sham. n = 66720 work/rest segments (derived from 7776 trials of 81 participants); data presented as mean values; error bars depict 95% confidence intervals at the segment level; dots depict condition means per participant. Source data are provided as a Supplementary Source Data file.

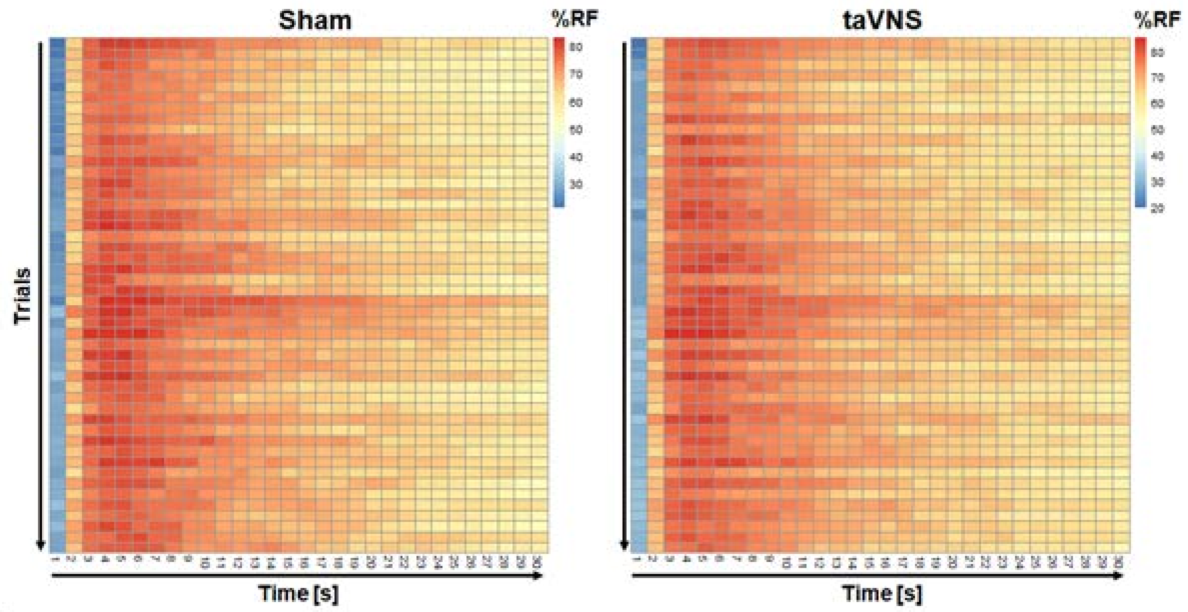

Supplementary Figure 3. Similar temporal profiles of effort over time for both stimulation conditions. Although effort was slightly higher overall for taVNS, there was no taVNS-induced change in the timing of work and rest phases apparent at a group level. To depict the heat map, time series data from n = 81 participants was binned by second and averaged for each condition. Warmer colors indicate higher values of relative frequency (%RF). Source data are provided as a Supplementary Source Data file.

Supplementary Table 1. Model output predicting invigoration

Final estimation of fixed effects:

Fixed Effect	Coefficient	Standard error	*t*-ratio	Approx. *d.f.*	*p*-value
For INTRCPT1, π_0					
INTRCPT2, β_{00}	55.316212	1.708055	32.386	78	<0.001
ORDER, β_{01}	5.584970	3.429248	1.629	78	0.107
STIMSIDE, β_{02}	-2.409802	3.416679	-0.705	78	0.483
For STIMCOND slope, π_1					
INTRCPT2, β_{10}	2.929325	0.995468	2.943	78	0.004
ORDER, β_{11}	-7.955717	1.998593	-3.981	78	<0.001
STIMSIDE, β_{12}	0.132041	1.991268	0.066	78	0.947
For FOOD slope, π_2					
INTRCPT2, β_{20}	-0.630546	0.824221	-0.765	78	0.447
ORDER, β_{21}	2.283221	1.654782	1.380	78	0.172
STIMSIDE, β_{22}	-0.056768	1.648716	-0.034	78	0.973
For REWM slope, π_3					
INTRCPT2, β_{30}	5.787697	1.235262	4.685	78	<0.001
ORDER, β_{31}	-1.608665	2.480027	-0.649	78	0.518
STIMSIDE, β_{32}	2.791219	2.470937	1.130	78	0.262
For CDIFF slope, π_4					
INTRCPT2, β_{40}	-2.437550	0.747349	-3.262	78	0.002
ORDER, β_{41}	0.740563	1.500448	0.494	78	0.623
STIMSIDE, β_{42}	-0.585218	1.494948	-0.391	78	0.697
For I_DRM slope, π_5					
INTRCPT2, β_{50}	0.071280	0.631970	0.113	78	0.910
ORDER, β_{51}	0.855155	1.268801	0.674	78	0.502
STIMSIDE, β_{52}	0.788098	1.264150	0.623	78	0.535
For I_SDIFF slope, π_6					
INTRCPT2, β_{60}	-0.614464	0.648915	-0.947	78	0.347
ORDER, β_{61}	1.621488	1.302822	1.245	78	0.217
STIMSIDE, β_{62}	-0.582364	1.298047	-0.449	78	0.655
For I_SREWM slope, π_7					
INTRCPT2, β_{70}	-0.334178	0.837629	-0.399	78	0.691
ORDER, β_{71}	-2.688585	1.681701	-1.599	78	0.114
STIMSIDE, β_{72}	-1.997746	1.675537	-1.192	78	0.237
For I_SFOOD slope, π_8					
INTRCPT2, β_{80}	1.325404	0.663475	1.998	78	0.049
ORDER, β_{81}	1.374944	1.332053	1.032	78	0.305
STIMSIDE, β_{82}	-2.816759	1.327171	-2.122	78	0.037
For I_SDRM slope, π_9					
INTRCPT2, β_{90}	0.170504	0.654726	0.260	78	0.795
ORDER, β_{91}	1.538027	1.314488	1.170	78	0.246
STIMSIDE, β_{92}	0.358150	1.309670	0.273	78	0.785

Note: n = 7776 trials of 81 participants. Statistics refer to two-sided t-contrasts of the mixed-effects model (no adjustments for multiple comparisons). Source data are provided as a Supplementary Source Data file.

Supplementary Table 2. Model output predicting effort maintenance

Final estimation of fixed effects:

Fixed Effect	Coefficient	Standard error	*t*-ratio	Approx. *d.f.*	*p*-value
For INTRCPT1, π_0					
INTRCPT2, β_{00}	65.156338	1.678245	38.824	78	<0.001
ORDER, β_{01}	-3.061905	3.369400	-0.909	78	0.366
STIMSIDE, β_{02}	-8.021054	3.357050	-2.389	78	0.019
For STIMCOND slope, π_1					
INTRCPT2, β_{10}	1.206855	0.703702	1.715	78	0.090
ORDER, β_{11}	-5.823920	1.412817	-4.122	78	<0.001
STIMSIDE, β_{12}	-0.518150	1.407638	-0.368	78	0.714
For FOOD slope, π_2					
INTRCPT2, β_{20}	-0.678189	0.901172	-0.753	78	0.454
ORDER, β_{21}	1.165683	1.809277	0.644	78	0.521
STIMSIDE, β_{22}	3.429187	1.802646	1.902	78	0.061
For REWM slope, π_3					
INTRCPT2, β_{30}	9.175745	1.294282	7.089	78	<0.001
ORDER, β_{31}	6.961399	2.598521	2.679	78	0.009
STIMSIDE, β_{32}	4.907710	2.588996	1.896	78	0.062
For CDIFF slope, π_4					
INTRCPT2, β_{40}	-6.712711	1.007945	-6.660	78	<0.001
ORDER, β_{41}	-3.372370	2.023643	-1.666	78	0.100
STIMSIDE, β_{42}	-3.022259	2.016226	-1.499	78	0.138
For I_DRM slope, π_5					
INTRCPT2, β_{50}	2.081447	0.536200	3.882	78	<0.001
ORDER, β_{51}	2.693999	1.076525	2.502	78	0.014
STIMSIDE, β_{52}	0.409628	1.072580	0.382	78	0.704
For I_SDIFF slope, π_6					
INTRCPT2, β_{60}	0.195107	0.320859	0.608	78	0.545
ORDER, β_{61}	-1.254500	0.644187	-1.947	78	0.055
STIMSIDE, β_{62}	0.597356	0.641826	0.931	78	0.355
For I_SREWM slope, π_7					
INTRCPT2, β_{70}	-0.031552	0.377138	-0.084	78	0.934
ORDER, β_{71}	-0.690027	0.757178	-0.911	78	0.365
STIMSIDE, β_{72}	-0.815119	0.754403	-1.080	78	0.283
For I_SFOOD slope, π_8					
INTRCPT2, β_{80}	0.065833	0.358979	0.183	78	0.855
ORDER, β_{81}	-0.191332	0.720720	-0.265	78	0.791
STIMSIDE, β_{82}	-0.933742	0.718078	-1.300	78	0.197
For I_SDRM slope, π_9					
INTRCPT2, β_{90}	-0.104746	0.247611	-0.423	78	0.673
ORDER, β_{91}	-0.436043	0.497126	-0.877	78	0.383
STIMSIDE, β_{92}	-0.435450	0.495304	-0.879	78	0.382

Note: n = 7776 trials of 81 participants. Statistics refer to two-sided t-contrasts of the mixed-effects model (no adjustments for multiple comparisons). Source data are provided as a Supplementary Source Data file.

Supplementary Table 3. Average parameter estimates from the cost-accumulation model

	Sham			taVNS-induced changes		
	M	95% CI	BF_{10}	*M*	95% CI	BF_{10}
A_{REW}	-0.06	-0.25 - 0.02	0.05	0.005	-0.04 - 0.05	0.020
SE_{MEAN}	0.18	0.15 - 0.20	>100	0.004	-0.006 - 0.020	0.005
SE_{DIFF}	0.02	-0.01 - 0.03	0.04	0.001	-0.005 - 0.007	0.004
SR_{MEAN}	1.51	1.31 - 1.74	>100	0.004	-0.07 - 0.09	< 001
SR_{REW}	0.50	0.36 - 0.66	>100	-0.004	-0.11 - 0.12	<.001

Note: *M* = mean, CI = Credible interval, BF_{10} = Bayes factor in favor of the alternative hypothesis, BF for all parameters were determined using the Savage Dickey density ratio method. BF < 1 can be interpreted as evidence for the null hypothesis (parameter value = 0), while BF > 1 can be interpreted as evidence for the alternative hypothesis (parameter value ≠ 0). Source data are provided as a Supplementary Source Data file.

Supplementary References

1. Organization WH. Waist circumference and waist-hip ratio: report of a WHO expert consultation, Geneva, 8-11 December 2008. (2011).

2. Watson D, Clark LA, Tellegen A. Development and validation of brief measures of positive and negative affect: the PANAS scales. *Journal of personality and social psychology* **54**, 1063 (1988).

3. Meyniel F*, et al.* A specific role for serotonin in overcoming effort cost. *Elife* **5**, (2016).

4. Meyniel F, Safra L, Pessiglione M. How the brain decides when to work and when to rest: dissociation of implicit-reactive from explicit-predictive computational processes. *PLoS Comput Biol* **10**, e1003584 (2014).

5. Meyniel F, Sergent C, Rigoux L, Daunizeau J, Pessiglione M. Neurocomputational account of how the human brain decides when to have a break. *Proc Natl Acad Sci U S A* **110**, 2641-2646 (2013).
