# Supplementary material for: Vagus nerve stimulation boosts the drive to work for rewards
Source: Nat Commun. 2020 Jul 16;11:3555. doi: 10.1038/s41467-020-17344-9 (PMC7366927; doi:10.1038/s41467-020-17344-9)
Supplement: Supplementary file 3 — Reporting Summary [file 41467_2020_17344_MOESM3_ESM.pdf]

nature research

Corresponding author(s): Nils B. Kroemer

Last updated by author(s): 05/05/2020

Reporting Summary

Nature Research wishes to improve the reproducibility of the work that we publish. This form provides structure for consistency and transparency in reporting. For further information on Nature Research policies, see Authors & Referees and the Editorial Policy Checklist.

Statistics

For all statistical analyses, confirm that the following items are present in the figure legend, table legend, main text, or Methods section.

n/a	Confirmed	
☐	☒	The exact sample size (*n*) for each experimental group/condition, given as a discrete number and unit of measurement
☐	☒	A statement on whether measurements were taken from distinct samples or whether the same sample was measured repeatedly
☐	☒	The statistical test(s) used AND whether they are one- or two-sided<br>*Only common tests should be described solely by name; describe more complex techniques in the Methods section.*
☐	☒	A description of all covariates tested
☐	☒	A description of any assumptions or corrections, such as tests of normality and adjustment for multiple comparisons
☐	☒	A full description of the statistical parameters including central tendency (e.g. means) or other basic estimates (e.g. regression coefficient) AND variation (e.g. standard deviation) or associated estimates of uncertainty (e.g. confidence intervals)
☐	☒	For null hypothesis testing, the test statistic (e.g. *F*, *t*, *r*) with confidence intervals, effect sizes, degrees of freedom and *P* value noted<br>*Give P values as exact values whenever suitable.*
☐	☒	For Bayesian analysis, information on the choice of priors and Markov chain Monte Carlo settings
☐	☒	For hierarchical and complex designs, identification of the appropriate level for tests and full reporting of outcomes
☐	☒	Estimates of effect sizes (e.g. Cohen's *d*, Pearson's *r*), indicating how they were calculated

Our web collection on statistics for biologists contains articles on many of the points above.

Software and code

Policy information about availability of computer code

Data collection	The task was presented using Psychophysics toolbox v3 (Brainard, 1997; Kleiner et al., 2007) in MATLAB v2017a.
Data analysis	Mixed-effects analyses were conducted with HLM v7 (Raudenbush, Bryk, Cheong, Congdon, & Du Toit, 2011) and lmerTest in R (Kuznetsova, Brockhoff, & Christensen, 2017). To determine the evidence provided by our results, we calculated corresponding BFs based on order-corrected ordinary least squares (OLS) estimates of all stimulation effects using the default Cauchy prior set to r = .707 as implemented in JASP v0.9 (JASP team, 2019). We also conducted a prior robustness analysis and changes in the prior would not have led to differences in evidential conclusions. Effort data was processed with MATLAB vR2017-2019a and SPSS v24. Results were plotted with R v3.4.0 (R Core Team, 2017).

For manuscripts utilizing custom algorithms or software that are central to the research but not yet described in published literature, software must be made available to editors/reviewers. We strongly encourage code deposition in a community repository (e.g. GitHub). See the Nature Research guidelines for submitting code & software for further information.

Data

Policy information about availability of data

All manuscripts must include a data availability statement. This statement should provide the following information, where applicable:

- Accession codes, unique identifiers, or web links for publicly available datasets
- A list of figures that have associated raw data
- A description of any restrictions on data availability

Trial-based behavioral data that was used to conduct all analyses will be made publicly available on OSF upon publication of the manuscript. Figures 2-6 are associated with raw data. Source file:
https://osf.io/58r3c/?view_only=5d1ccee7d67b464bb6f40ebe7ebc844b

Field-specific reporting

Please select the one below that is the best fit for your research. If you are not sure, read the appropriate sections before making your selection.

☐ Life sciences ☒ Behavioural & social sciences ☐ Ecological, evolutionary & environmental sciences

For a reference copy of the document with all sections, see nature.com/documents/nr-reporting-summary-flat.pdf

Behavioural & social sciences study design

All studies must disclose on these points even when the disclosure is negative.

Study description	Randomized single-blind crossover experimental study, transcutaneous auricular vagus nerve stimulation (taVNS) as experimental manipulation. Quantitative data consist of timeseries data (effort exertion over time) and subjective ratings (via visual analog scales, VAS).
Research sample	The research sample consists of volunteers from the area of Tübingen, Germany, that were recruited via university Email lists and advertisements at public places and on social media. Participants were physically and mentally healthy, German speaking, and right-handed, as determined by a telephone interview (48 women; M_age= 25.3 years ± 3.8; M_BMI= 23.0 kg per/ m^2 ± 2.95; 17.9 - 30.9).
Sampling strategy	Sample size weas determined based on effect-size estimations. Proposed minimal sample size of N = 40 per stimulation condition would allow to assess medium-sized effects (Cohen's f = .20, dz ~ .40) with sufficiently high power (1-β = .79), given a moderate reliability of behavioral measures (r12 = .60). A higher reliability (as we observed) increases power beyond this lower-bound estimate,
Data collection	Data collection (effort task data, ratings via visual analog scales) were completed using computerized paradigms coded in Psychtoolbox, MATLAB. Experimental sessions were conducted in a randomized, single-blind crossover design. Blinding of experimenters was not possible because all of them were trained psychologists, neuroscientists, or physicians so that they knew about the vagal innervation of the ear.
Timing	For the subsample receiving stimulation at the left ear, we started data collection in August 2017 and ended in May 2018. For the subsample receiving stimulation at the right ear, we started data collection in November 2018 and ended in June 2019.
Data exclusions	For the current analysis, 4 participants had to be excluded (the n=3: who did not finish the second experimental session, see "Non-participation", n=1: was assigned an incorrect maximum of button press frequency precluding comparison of the two sessions) leading to a total sample size of N = 81.
Non-participation	n=3 participants were categorized as drop-outs since they did not finish the second experimental session, for example due to sick leave
Randomization	According to the randomized crossover study design, each participant underwent the experimental procedure twice (taVNS and sham), and randomization of the order of stimulation conditions was preset and determined with MATLAB's datasample function. Participants were allocated randomly to first-session stimulation condition. To control for covariates (e.g., order of stimulation conditions) we added them as group mean centered regressors to our mixed-effects models.

Reporting for specific materials, systems and methods

We require information from authors about some types of materials, experimental systems and methods used in many studies. Here, indicate whether each material, system or method listed is relevant to your study. If you are not sure if a list item applies to your research, read the appropriate section before selecting a response.

Materials & experimental systems

n/a	Involved in the study	
☒	☐	Antibodies
☒	☐	Eukaryotic cell lines
☒	☐	Palaeontology
☒	☐	Animals and other organisms
☐	☒	Human research participants
☒	☐	Clinical data

Methods

n/a	Involved in the study	
☒	☐	ChIP-seq
☒	☐	Flow cytometry
☒	☐	MRI-based neuroimaging

Human research participants

Policy information about studies involving human research participants

Population characteristics	Participants were physically and mentally healthy, German speaking, and right-handed, as determined by a telephone interview (48 women; Mage= 25.3 years ± 3.8; MBMI= 23.0 kg/m2 ± 2.95; 17.9 - 30.9).

Recruitment	Participants were recruited via university mailing lists, flyers, hand-outs, and word of mouth. No potential self-selection biases known for the recruitment of participants.
Ethics oversight	The study was approved by the institutional review board of the Faculty of Medicine, University of Tübingen (#235/2017BO1)

Note that full information on the approval of the study protocol must also be provided in the manuscript.
